# Mitochondrial Dysfunction Unravels the Potential Molecular Link Between Night Shift Work‐Related Circadian Disruption and Elevated Blood Pressure in Human and Mouse Models

**DOI:** 10.1002/advs.202520318

**Published:** 2026-06-19

**Authors:** Zhaoqiang Jiang, Yifan Dou, Yingnan Lei, Shuang Liu, Jialin Zhou, Jiaping Li, Huadong Xu, Lingfang Feng, Yongxin Li, Chuyan Zhang, Tao Li, Jianfei Wang, Xing Zhang, Xinran Wang, Luyao Liu, Jingjing Zhang, Jianlin Lou

**Affiliations:** ^1^ School of Public Health Hangzhou Medical College Hangzhou Zhejiang Province China; ^2^ School of Medicine and the First Affiliated Hospital Huzhou University Huzhou Zhejiang Province China; ^3^ Department of Environmental and Occupational Health Jinshan District Center for Disease Control and Prevention Shanghai China; ^4^ The Third People's Hospital of Tongxiang City Jiaxing Zhejiang Province China

**Keywords:** blood pressure elevation, circadian rhythem, mitochondrial dysfunction, night shift work

## Abstract

This study demonstrates that circadian rhythm disruption induced by night‐shift work is associated with elevated blood pressure in both murine models and human subjects. By integrating rodent and human epidemiological data, we identify mitochondrial dysfunction as the principal mechanistic contributor to this relationship. In mice, data were obtained at 4 and 10 weeks, with sampling performed at both ZT0 and ZT12. Simulated night shift work elevates blood pressure, induces circadian gene dysregulation (e.g., PER1, BMAL1), and precipitates mitochondrial dysfunction and oxidative stress. These findings are corroborated in human night shift workers, where exposure duration is quantitatively associated with increased systolic and diastolic blood pressure and concomitant dysregulation of circadian and mitochondrial markers. Furthermore, the expression levels of these markers correlate with both night shift work exposure and blood pressure. Collectively, the results establish mitochondrial dysfunction as a critical pathway linking circadian disruption to an elevated blood‐pressure phenotype, offering new insights into the cardiovascular consequences of shift work.

## Graphical abstract

This diagram illustrates that night shift work disrupts circadian clock genes (like CLOCK, BMAL1) in both humans and mice. This disruption leads to mitochondrial dysfunction (imbalanced fusion/fission proteins) and increased oxidative stress, which is identified as the primary mechanism ultimately causing elevated blood pressure.

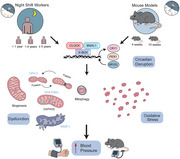

## 1 Introduction

With the increasing societal demands and growing occupational specialization, night shift work has become increasingly prevalent in industrialized societies [1]. This occupational pattern can disrupt circadian rhythm, contributing to diverse pathological disorders, including neurologic dysfunction, psychiatric dysregulation, metabolic disorders, and cardiovascular diseases [2]. Notably, accumulating evidence indicates that variations in shift patterns can affect blood pressure regulation in shift workers [3, 4]. Night shift work has been identified as a predominant disruptor of circadian physiology, causing substantial dysregulation of core circadian gene expression among workers. Experimental animal studies further demonstrate that dysregulation of these circadian genes directly affects blood pressure regulation [5, 6]. Emerging insights suggest that circadian rhythm disrupts blood pressure through an imbalance in the autonomic nervous system or dysregulated sodium retention [7, 8]. Although the association between circadian disruption and hypertension has been extensively studied, the precise molecular and physiological mechanisms underlying this relationship require further elucidation.

Aberrant mitochondrial function, particularly an impaired mitochondrial fission‐fusion balance (mitochondrial dynamics), represents a key mechanistic driver of disease pathogenesis [9]. Night shift work disrupts the circadian rhythm, thereby impairing mitochondrial function [10]. This circadian disruption, driven by core circadian gene dysregulation, can trigger mitochondrial alterations [11, 12], which are characterized by reduced mitochondrial content and volumetric density, abnormal morphological changes, disturbed mitochondrial bioenergetics, and dynamic activation [13, 14]. Mitochondrial dysfunction may contribute to hypertension pathogenesis, potentially through altered expression of mitochondrial‐associated proteins [15]. Although circadian disruption has been well‐established as a precursor to hypertension, the underlying pathophysiological mechanisms remain elusive. Mitochondria and their dynamic regulation are essential for maintaining cellular oxidative stress balance [16]. The mechanistic interplay between circadian rhythm disruption, mitochondrial dysfunction, and oxidative stress in shift work‐associated blood pressure elevation requires further investigation.

In this study, we investigated the association between night shift work‐induced circadian disruption and blood pressure regulation using complementary rodent models and human studies. The research further systematically explored the underlying pathogenic mechanisms, with particular focus on the roles of mitochondrial dysfunction and oxidative stress in circadian disruption‐driven blood pressure elevation.

## 2 Results

### 2.1 Circadian Disruption Leads to Increased Blood Pressure in Night Shift Simulation Mice

**FIGURE 1:**
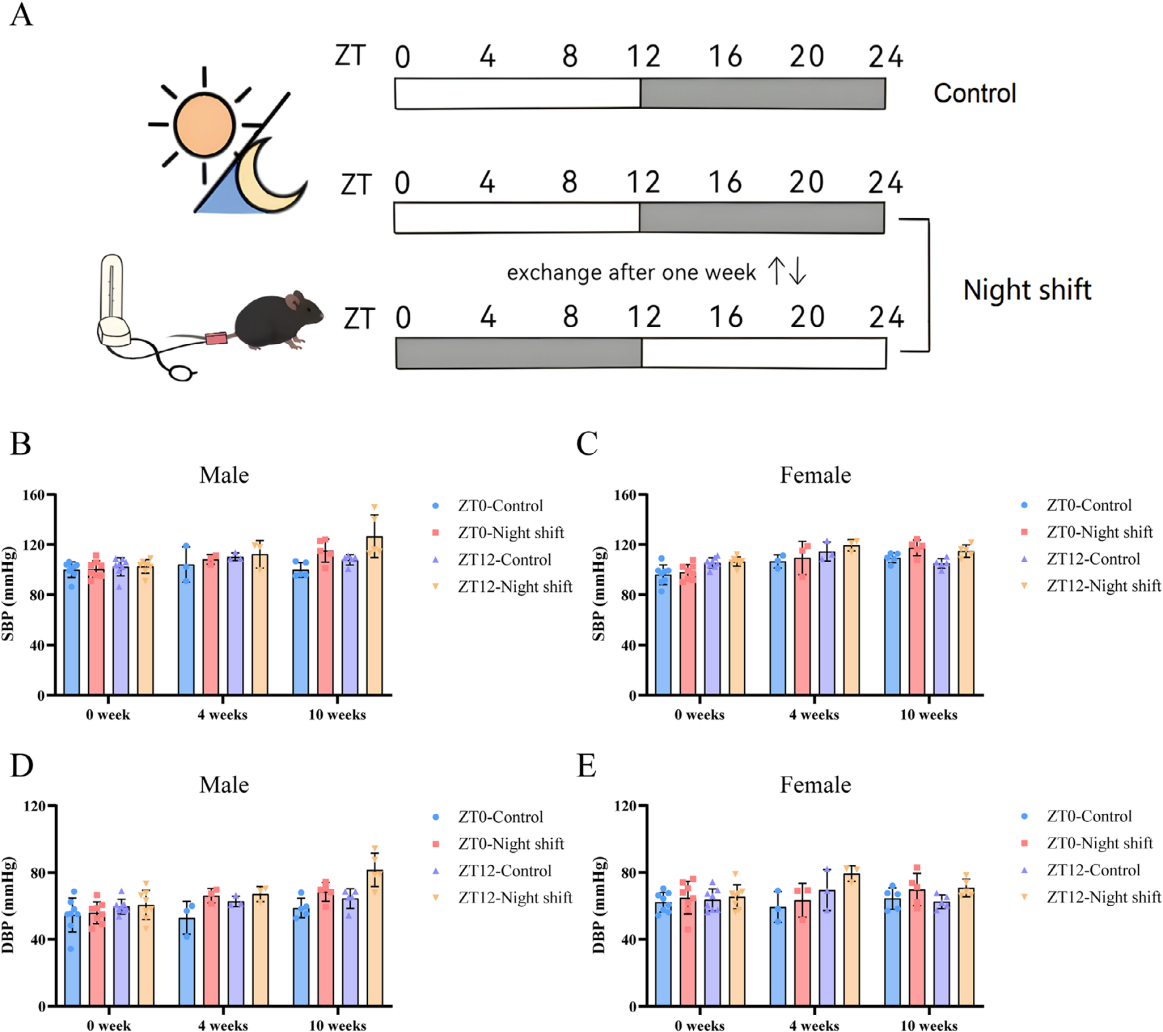
The impact of night shift work simulation on blood pressure. Night shift: night shift work simulation. SBP: systolic blood pressure; DBP: diastolic blood pressure. (A) Experimental design of the mouse model of night shift work simulation. (B, C) SBP level comparison in male (B) and female (C) mice between control and night shift conditions at ZT0 and ZT12 at 4 weeks (*n* = 3) and 10 weeks (*n* = 5). (D, E) DBP level comparison in male (D) and female (E) mice between control and night shift conditions at ZT0 and ZT12 at 4 weeks (*n* = 3) and 10 weeks (*n* = 5). Data were presented as mean ± SD. Statistical analysis was performed using two‐way ANOVA followed by estimated marginal means (EMM) comparisons. At 4 weeks, the interaction for SBP in males: *P*_treatment × time_ = 0.856; main effects for SBP in males: *P*_treatment_ = 0.553 and *P*_time_ = 0.338; the interaction for DBP in males: *P*_treatment × time_ = 0.237; main effects for DBP in males: *P*_treatment_ = 0.037 and *P*_time_ = 0.164. The interaction for SBP in females: *P*_treatment × time_ = 0.832; main effects for SBP in females: *P*_treatment_ = 0.410 and *P*_time_ = 0.082; the interaction for DBP in females: *P*_treatment × time_ = 0.606; main effects for DBP in females: *P*_treatment_ = 0.219 and *P*_time_ = 0.035. At 10 weeks, the interaction for SBP in males: *P*_treatment × time_ = 0.287; main effects for SBP in males: *P*_treatment_ *=* 0.004 and *P*_time_ = 0.009; the interaction for DBP in males: *P*_treatment × time_ = 0.065; main effects for DBP in males: *P*_treatment_ = 0.009 and *P*_time_ = 0.006. The interaction for SBP in females: *P*_treatment × time_ = 0.667; main effects for SBP in females: *P*_treatment_ = 0.001 and *P*_time_ = 0.118; the interaction for DBP in females: *P*_treatment × time_ = 0.646; main effects for DBP in females: *P*_treatment_ = 0.033 and *P*_time_ = 0.875).

After 4 weeks of simulated night‐shift exposure (Figure 1A), significantly elevated DBP was observed in the night‐shift group compared to the control group in male mice (*p* = 0.037), with both SBP and DBP showing an upward trend. At 10 weeks, the night shift group had significantly higher SBP and DBP than the control group in male mice (*p* = 0.004 and 0.009, respectively) and female mice (*p* = 0.001 and 0.033, respectively). Simulated night‐shift exposure also led to greater body weight gain in both male and female mice compared to the control group (Figure S1).

### 2.2 Night Shift Work Simulation Affects Circadian Gene Expression Profiles in the Mice's Heart

**FIGURE 2:**
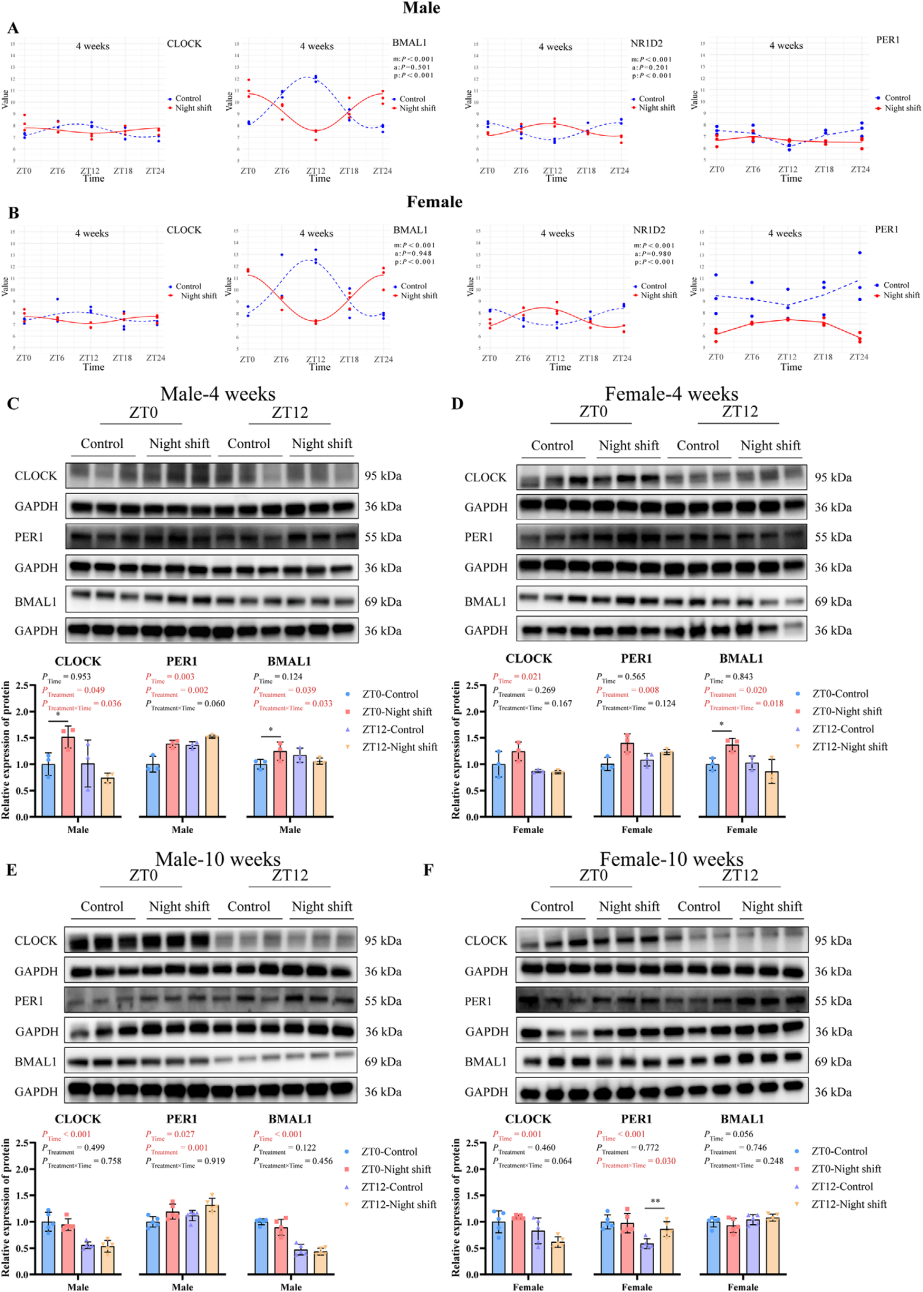
The impact of night shift work simulation on circadian clock genes. Night shift: night shift work simulation. m: mesor. a: amplitude. p: phase. (A, B) mRNA expression of circadian markers in the myocardium of male (A) and female (B) mice at 5 timepoints: comparison between control and night‐shift group after 4 weeks of simulated night shift (*n* = 3 per time point). (C, D) Protein expression levels of circadian markers in male (C) and female (D) mice at ZT0 and ZT12: comparison between control and night shift groups after 4 weeks (*n* = 3) of simulated night shift exposure. (E, F) Protein expression levels of circadian markers in male (E) and female (F) mice at ZT0 and ZT12: comparison between control and night shift groups after 10 weeks (*n* = 3) of simulated night shift exposure. Due to rhythmicity loss in the time‐dependent expression curves of CLOCK and PER1, valid amplitude and peak timing data are unavailable, precluding statistical comparisons between groups. Data presented as mean ± SD. Statistical analysis was performed using two‐way ANOVA followed by EMM comparisons. ^*^ *p* < 0.05; ^**^ *p* < 0.01.

Next, we compared mRNA expression levels (4‐week time point) and protein expression levels (4‐ and 10‐week time points) between the night‐shift work simulation group and the control group. At 4 weeks, the simulation group exhibited a disrupted circadian rhythm compared to the control group, with altered 24‐h expression patterns of cardiac circadian genes (Figure 2A,B; Figure S2). A significant treatment × time interaction was observed for CLOCK and BMAL1 in male mice (*p* = 0.036 and 0.033, respectively), while a significant treatment × time interaction was observed for BMAL1 in female mice (*p* = 0.018). Two‐way ANOVA revealed a significant main effect of treatment on CLOCK, PER1, and BMAL1 protein expression in male mice (*p* = 0.049, 0.002, and 0.039, respectively) and a significant main effect of treatment on PER1 and BMAL1 protein expression in female mice (*p* = 0.008 and 0.020, respectively). At ZT0, night shift simulation induced significant circadian disruptions: CLOCK and BMAL1 protein expression were increased in males (*p* = 0.049 and 0.039, respectively; Figure 2C) and BMAL1 protein expression was up‐regulated in females (*p* = 0.020; Figure 2D). At 10 weeks, circadian disruption persisted in both male and female mice. A significant treatment × time interaction was observed for PER1 in female mice (*p* = 0.030). Two‐way ANOVA showed a significant main effect of treatment on PER1 protein expression in male mice (*p* = 0.001). At ZT12, PER1 protein expression was significantly up‐regulated in the female simulation groups compared to the control group (*p* = 0.007; Figure 2F; Figure S3B). Original Western blot images are provided in Figure S4.

### 2.3 Circadian Disruption Caused Mitochondrial Dysfunction in Mice

**FIGURE 3:**
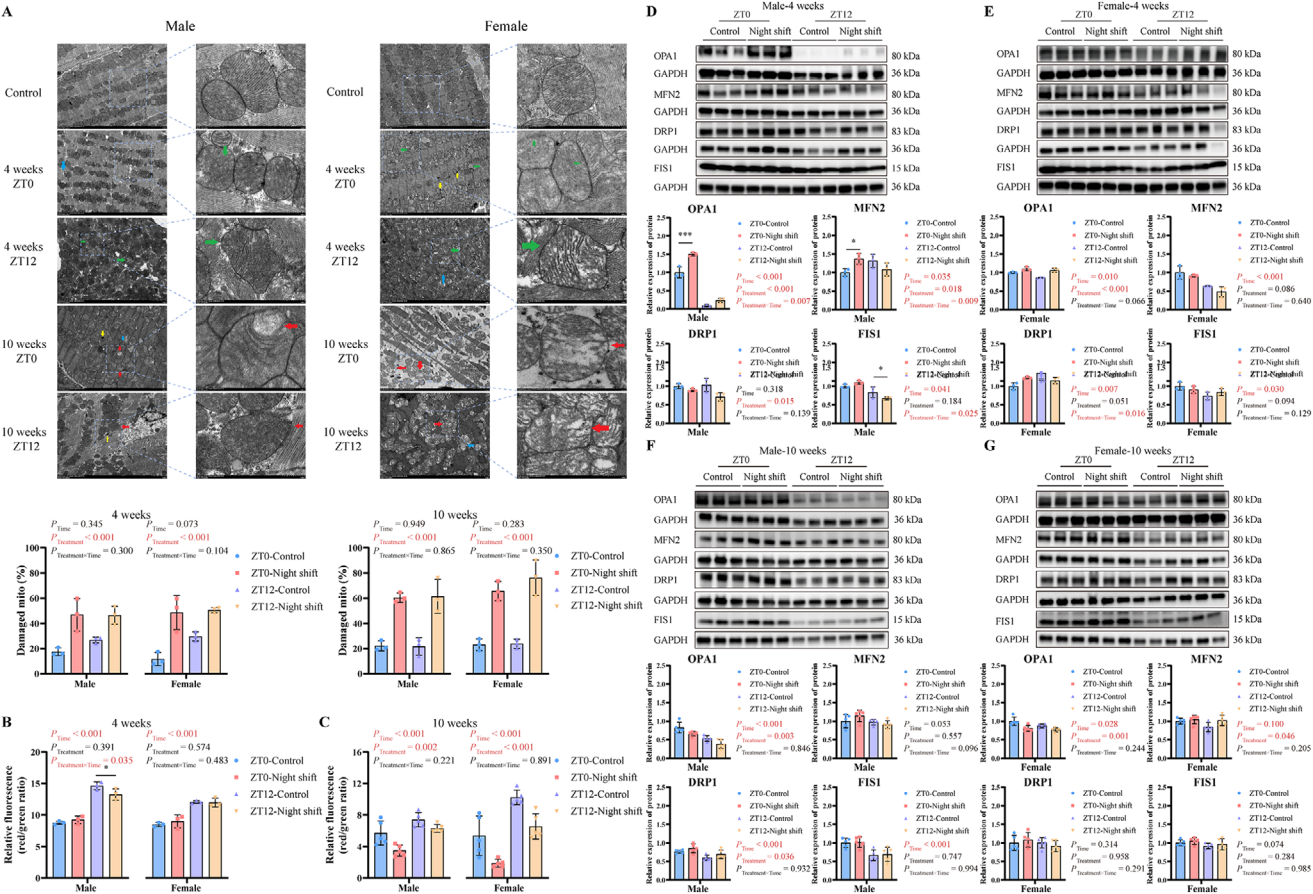
Mitochondrial dysfunction induced by night‐shift work simulation. Night shift: night shift work simulation. (A) Representative transmission electron micrographs of cardiomyocyte mitochondria from control and night‐shift mice. Yellow arrow: lysosome granules; blue arrow: lipid droplets accumulation; green arrow: mitochondria with blurred outer membrane boundaries and slight rupture; red arrow: severe mitochondrial damage, including membrane rupture, disrupted cristae (fractures, folding, loose arrangement, complete loss, and matrix vacuolization). Scale bar: 2.0 µm and 500 nm. The percentage of damaged mitochondria is shown. (B, C) Mitochondrial membrane potential in male mice and females at ZT0 and ZT12 after 4 weeks (B, *n* = 3) and 10 weeks (C, *n* = 5). D–E) Mitochondrial marker protein expression in male (D) and female (E) mice at ZT0 and ZT12 after 4 weeks (*n* = 3). F–G) Mitochondrial marker protein expression in male (F) and female (G) mice at ZT0 and ZT12 after 10 weeks (*n* = 3). Data presented as mean ± SD. Statistical analysis was performed using two‐way ANOVA followed by EMM comparisons. ^*^ *p* < 0.05; ^***^ *p* < 0.001.

The night‐shift work simulation group demonstrated significant changes in mitochondrial function. Transmission electron microscopy (TEM) revealed that 4 weeks of night‐shift work simulation induced distinct mitochondrial ultrastructural abnormalities, including blurred outer membranes, slight ruptures, matrix efflux, increased membrane density, indistinct cristae, reduced cristae numbers, and irregular arrangements. Quantitative analysis confirmed a significantly higher proportion of damaged mitochondria in the simulation group (*p* < 0.001; Figure 3A). Notably, female mice in the simulation group exhibited increased lysosomal particles and greater mitochondrial lipid droplet accumulation than the control group at both ZT0 and ZT12. These ultrastructural abnormalities progressively worsened with prolonged exposure. At 10 weeks, cardiomyocyte mitochondrial damage in the simulation group was severe at both ZT0 and ZT12, featuring ruptured outer membranes, blurred or disrupted cristae with folding, loose arrangements, or complete loss, and vacuolization of the mitochondrial matrix. Quantitative analysis again confirmed a significantly higher proportion of damaged mitochondria in the simulation group (*p* < 0.001, respectively; Figure 3A). Mitochondrial membrane potential in myocardial tissue was also measured. A decline in membrane potential was observed in night‐shift male mice at ZT12 after 4 weeks (*p* = 0.028; Figure 3B). At 4 weeks, two‐way ANOVA demonstrated significant treatment × time interactions in membrane potential in male mice (*p* = 0.035). At 10 weeks, significant main effects of treatment on membrane potential were detected in both male and female mice (*p* = 0.002 and *p* < 0.001, respectively; Figure 3C).

Western Blot analysis further validated significant time‐dependent alterations in mitochondrial dynamics‐related protein expression. At 4 weeks, significant treatment × time interactions were observed for OPA1, MFN2, and FIS1 in male mice (*p* = 0.007, 0.009, and 0.025, respectively). In addition, two‐way ANOVA showed significant main effects of treatment for OPA1, MFN2, and DRP1 in male mice (*p* < 0.001, *p* = 0.018, and 0.015, respectively). Significantly, night‐shift exposure significantly increased OPA1 and MFN2 protein expression in male mice at ZT0 (*p* < 0.001 and *p* = 0.018, respectively; Figure 3D). Conversely, it decreased FIS1 protein expression in male mice at ZT12 (*p* = 0.041). In female mice, a significant treatment × time interaction was found for DRP1 (*p* = 0.016). Furthermore, two‐way ANOVA also showed a significant main effect of treatment for OPA1 (*p* < 0.001). At 10 weeks, two‐way ANOVA found significant main treatment effects on OPA1 and DRP1 in male mice (*p* = 0.003 and 0.036, respectively). Similarly, treatment effects for OPA1 and MFN2 were also significant in female mice (*p* = 0.001 and 0.046, respectively).

We further assessed protein levels of markers for mitochondrial biogenesis, oxidative phosphorylation (OXPHOS), and mitophagy. At 4 weeks, significant two‐way interactions were observed for NDUFB8, PGC‐1, LC3 I, and P62 in male mice (*p* = 0.002, 0.047, 0.001, and *p* < 0.001, respectively). Specifically, male mice showed up‐regulation of LC3 I, PGC‐1, and P62 at ZT0 (*p* = 0.004, 0.025, and *p* < 0.001, respectively). Conversely, at ZT12, NDUFB8 and LC3 I expression decreased in male mice (*p* = 0.004 and 0.017). In contrast, in female mice, significant interactions were found for LC3 I and LC3 II (*p* = 0.002 and 0.012, respectively; Figure S5J). Female mice showed up‐regulation of LC3 I at ZT0 (*p* = 0.017), whereas both LC3 I and LC3 II expression were down‐regulated at ZT12 (*p* = 0.008 and 0.022, respectively).

At 10 weeks, a significant treatment × time interaction was observed for NDUFB8 in male mice (*p* = 0.029), while MTCO2 and PGC‐1 exhibited a significant treatment × time interaction in female mice (*p* = 0.023 and 0.001, respectively). NDUFB8 showed significant down‐regulation in male mice at ZT12 (*p* = 0.032; Figures S3E and S5C). For female mice, there was significant up‐regulation of PGC‐1 at ZT0 (*p* = 0.013) and down‐regulation at ZT12 (*p* = 0.008; Figures S3H and S5H).

### 2.4 Circadian Rhythm Disruption Affects Oxidative Stress Levels in Myocardial Tissue

**FIGURE 4:**
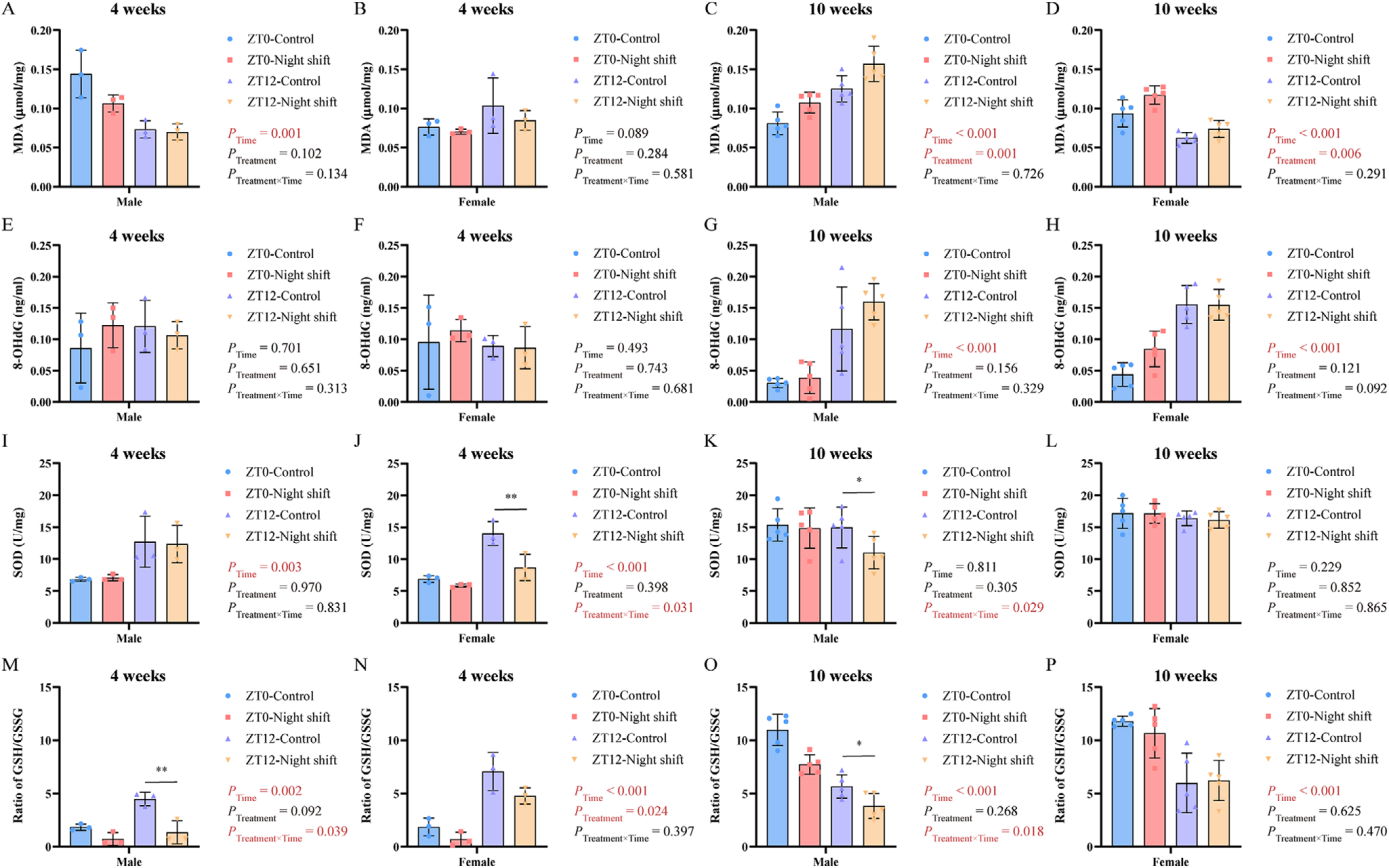
Night‐shift work simulation alters oxidative stress levels. Night shift: night shift work simulation. (A–D) MDA levels in myocardial tissues of control and night shift mice in male and female mice at 4 weeks (A and B, *n* = 3) and 10 weeks (C and D, *n* = 5). (E–H) 8‐OHdG levels in myocardial tissues of control and night shift male and female mice at 4 weeks (E and F, *n* = 3) and 10 weeks (G and H, *n* = 5). (I–L) SOD levels in myocardial tissues of control and night shift in male and female mice at 4 weeks (I and J, n = 3) and 10 weeks (K and L, *n* = 5). (M–P) GSH/GSSG ratio in myocardial tissues of control and simulated night shift in male and female mice at 4 weeks (M and N, *n* = 3) and 10 weeks (O and P, *n* = 5). Data presented as mean ± SD. Statistical analysis was performed using two‐way ANOVA followed by EMM comparisons. ^*^ *p* < 0.05; ^**^ *p* < 0.01.

To investigate the effects of night shifts on myocardial oxidative stress, we measured levels of malondialdehyde (MDA), 8‐hydroxydeoxyguanosine (8‐OHdG), superoxide dismutase (SOD), and glutathione / oxidized glutathione disulfide (GSH/GSSG) in mice after 4 and 10‐week simulated night‐shift exposures (Figure 4). The results showed significant oxidative stress in mice exposed to night shift conditions. At 4 weeks, two‐way ANOVA showed a significant treatment × time interaction for GSH/GSSG in male mice (*p* = 0.039). In males, the GSH/GSSG ratios decreased at ZT12 (*p =* 0.001). For female mice, there was a significant treatment × time interaction for SOD (*p* = 0.031). At ZT12, females showed a downward trend in SOD (*p =* 0.002). Female mice also exhibited a significant up‐regulation of GSH/GSSG (*p =* 0.024).

At 10 weeks, two‐way ANOVA found a significant treatment × time interaction for SOD and GSH/GSSG in male mice (*p =* 0.029 and 0.018). Night‐shift male mice had higher MDA than controls (*p* = 0.001). At ZT12, these mice also had lower SOD and GSH/GSSG than controls (*p* = 0.033 and 0.020). Female night‐shift mice showed increased MDA compared to controls (*p* = 0.006).

### 2.5 Circadian Disruption Links Night Shift Work to Altered Mitochondrial Dynamics and Elevated Blood Pressure in Mice

**FIGURE 5:**
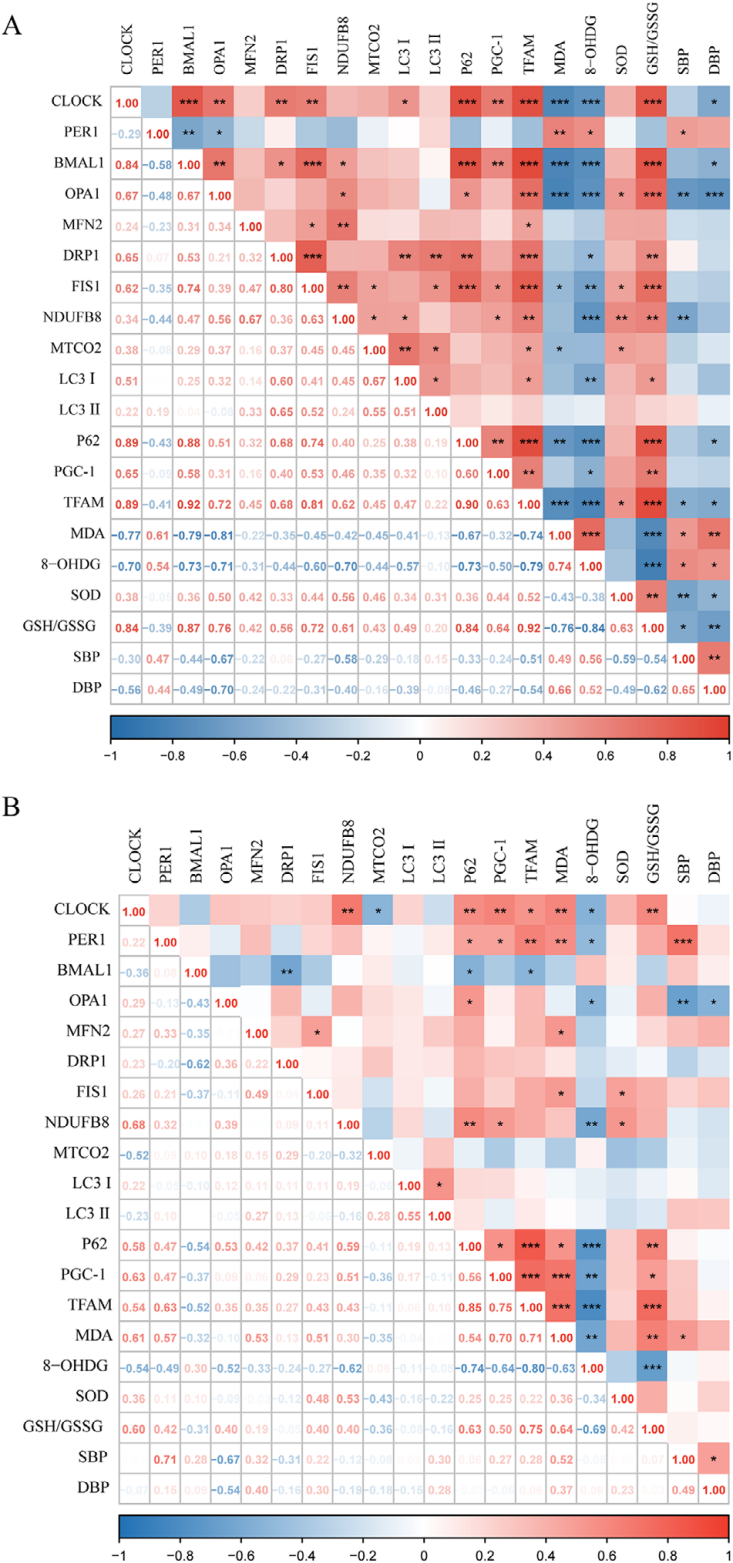
The impact of night‐shift work simulation on blood pressure and mitochondrial genes in a mouse model. (A, B) Pearson correlation analysis of blood pressure with protein expression profiles of circadian markers, mitochondrial markers, and oxidative stress markers in male (A, *n* = 10) and female (B, *n* = 10) mice. Numerical values indicate correlation coefficients. The numbers in the figure represent Pearson's correlation coefficients, with red and blue indicating positive and negative values, respectively. ^*^ *p* < 0.05; ^**^ *p* < 0.01; ^***^ *p* < 0.001.

A subsequent correlation analysis was conducted to systematically assess the integrative relationships among circadian clock protein expression profiles, mitochondrial‐related proteins, oxidative stress biomarkers, and blood pressure (Figure 5). Significant associations were observed between blood pressure and circadian clock gene expression: in males, PER1 showed a significant positive correlation with SBP (*r* = 0.47, *p* < 0.036, Figure 5A), CLOCK and BMAL1 indicated significant associations with DBP (*r* = −0.56, *p* = 0.011; *r* = −0.49, *p* = 0.027, respectively). Conversely, in females, positive correlations were observed between blood pressure and circadian clock protein expression, with PER1 showing a significant association (*r* = 0.71, *p* < 0.001; Figure 5B). Mitochondrial markers OPA1 showed negative correlations with SBP in males and females (*r* = −0.67, −0.67, −0.7, and −0.54, respectively; *p* = 0.001, 0.001, 0.001, and 0.014, respectively). Additionally, the mitochondrial markers NDUFB8 and P62 exhibited negative correlations with blood pressure in male mice (*r* = −0.58 and −0.46, respectively; *p* = 0.007 and 0.043, respectively). Additionally, PER1 protein levels were negatively correlated with OPA1 in males (*r* = −0.48, *p* = 0.031). In females, the BMAL1 protein level was negatively related to DRP1 (*r* = −0.62, *p* = 0.004). Furthermore, BMAL1 protein levels showed negative correlations with TFAM and P62 (*r* = −0.52 and −0.54, respectively; *p* = 0.018 and 0.013, respectively). Moreover, strong correlations between blood pressure and oxidative stress markers were exclusive to male mice (MDA and 8‐OHdG: *r* = 0.49, 0.56, 0.66, and 0.52, respectively; *p* = 0.028, 0.011, 0.002 and 0.018, respectively; SOD and GSH/GSSG ratio: *r* = −0.59, −0.54, −0.49 and −0.62, *p* = 0.006, 0.014, 0.03 and 0.003, respectively). MDA was also positively correlated with SBP in female mice (*r* = 0.52, *p* = 0.02).

### 2.6 Characteristics of Night Shift Workers

The study recruited 279 night shift workers. All night shift workers included in our study were engaged in rotating shift work. The demographic and clinical characteristics, including gender, age, education level, smoking status, alcohol consumption, BMI, SBP, and DBP, are shown in Table 1. Of the participants, 58.8% were female, and the overall median age was 41 (36–44) years. Subjects working for ≥5 years had higher SBP and DBP than those working for <1 year and 1–5 years (*p* < 0.001). Subjects working for ≥5 years were older than those in other groups (*p* < 0.001 and 0.012, respectively; Table 1).

**TABLE 1:** Characteristics of participants.

Characteristics	Duration of night shift work exposure (year)			*p*‐value
	<1 (*n* = 74)	1–5 (*n* = 104)	≥5 (*n* = 101)	
Gender^a^				0.875
Male	32 (43.2)	41 (39.4)	42.0 (41.6)	
Female	42 (56.8)	63 (60.4)	59.0 (58.4)	
Age (y)^b^	41 (37, 43.3)	38 (31, 41)^***^, ^###^	44.0 (39.5, 46)^*^	<0.001
Education level^a^				0.487
Below high school	59 (79.7)	82 (78.8)	87 (86.1)	
High school and above	15 (20.3)	22 (21.2)	14 (13.9)	
Having smoking habit^a^				0.394
No	52 (70.3)	82 (78.8)	78 (77.2)	
Yes	22 (29.7)	22 (21.2)	23 (22.8)	
Having drinking habit^a^				0.273
No	48 (64.9)	73 (70.2)	60 (59.4)	
Yes	26 (35.1)	31 (29.8)	41 (40.6)	
BMI^a^				0.524
Normal	44 (59.5)	64 (61.5)	68 (67.3)	
Overweight	30 (40.5)	40 (38.5)	33 (32.7)	
SBP (mmHg)^b^	124 (113.8, 137.3)	120 (109.3, 131)^###^	142 (121, 149)^***^	<0.001
DBP (mmHg)^b^	76.5 (70, 86)	74.5 (68, 81)^###^	84 (74, 91.5)^**^	<0.001

BMI: body mass index; SBP: systolic blood pressure; DBP: diastolic blood pressure.

^a^ The results are presented as a number (%);

^b^ The results are presented as *M* (*P*_25_, *P*_75_).

** compared to workers having <1 year of night shift work exposure, *p* < 0.01;

*** compared to workers having <1 year of night shift work exposure, *p* < 0.001;

^###^ compared to workers having 1–4 years of night shift work exposure, *p* < 0.001.

### 2.7 Circadian Disruption Is Related to Increased Blood Pressure in Night Shift Workers

**FIGURE 6:**
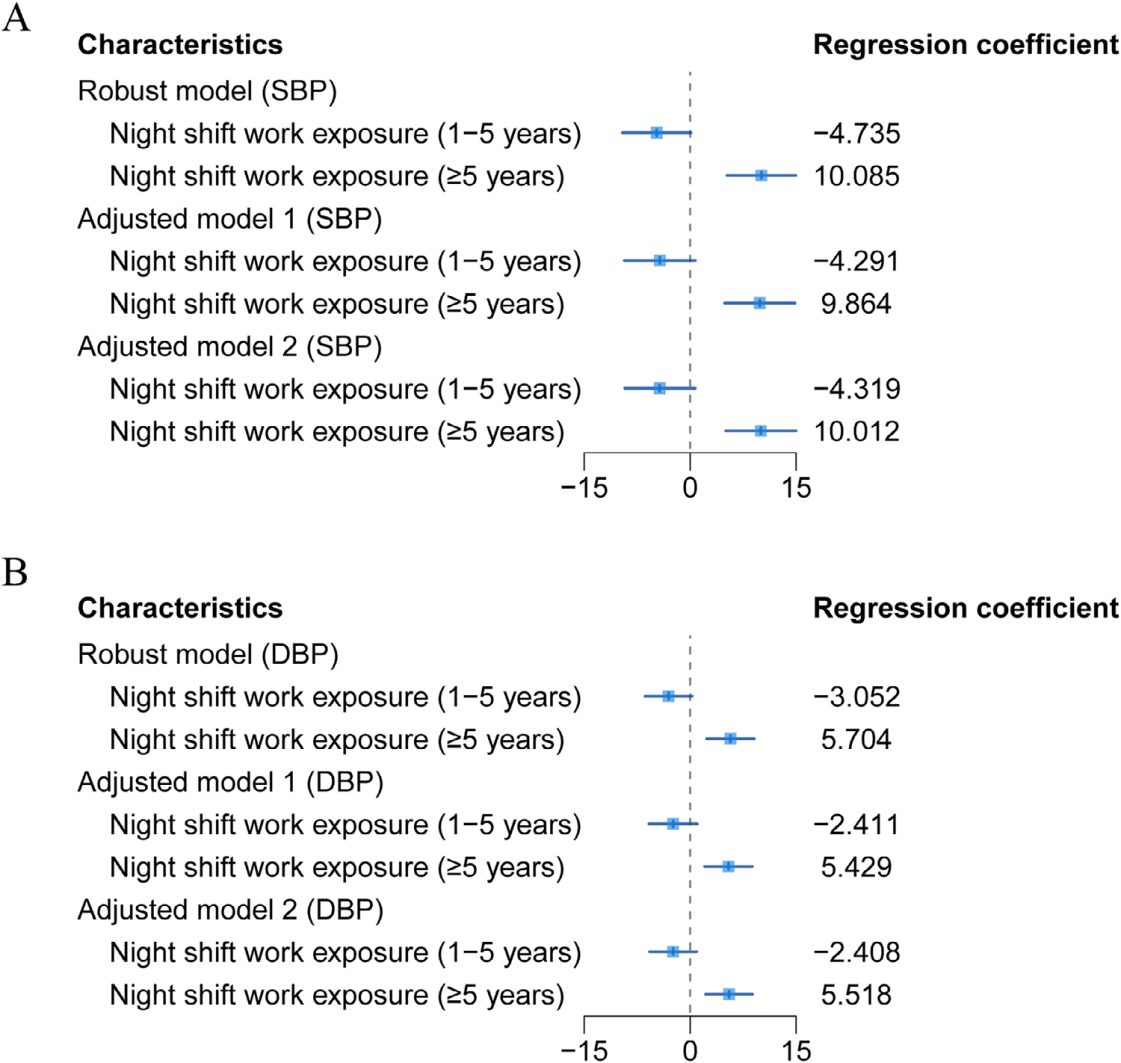
Correlation between night shift work exposure and blood pressure. NSWE: duration of night shift work exposure; SBP: systolic blood pressure; DBP: diastolic blood pressure. The adjusted Model 1 was adjusted by age and gender; the adjusted Model 2 was further adjusted by education level, smoking habit, drinking habit, and body mass index. (A) The association between workers' night shift work exposure and SBP. (B) The association between night shift work exposure and DBP.

The association between night shift work and blood pressure is presented in Figure 6. Multivariate linear regression analysis of the adjusted model demonstrated that workers with ≥5 years of night shift exposure exhibited significantly elevated risks compared to those with <1 year of exposure, with a 10.012‐fold increase in SBP (95% CI: 5.042–14.981) and a 5.518‐fold increase in DBP (95% CI: 2.216–8.819; Figure 6A,B), consistent with our mice model findings.

### 2.8 Circadian Disruption Links Night Shift Work to Altered Mitochondrial Dynamics and Elevated Blood Pressure in the Night Shift Workers

**FIGURE 7:**
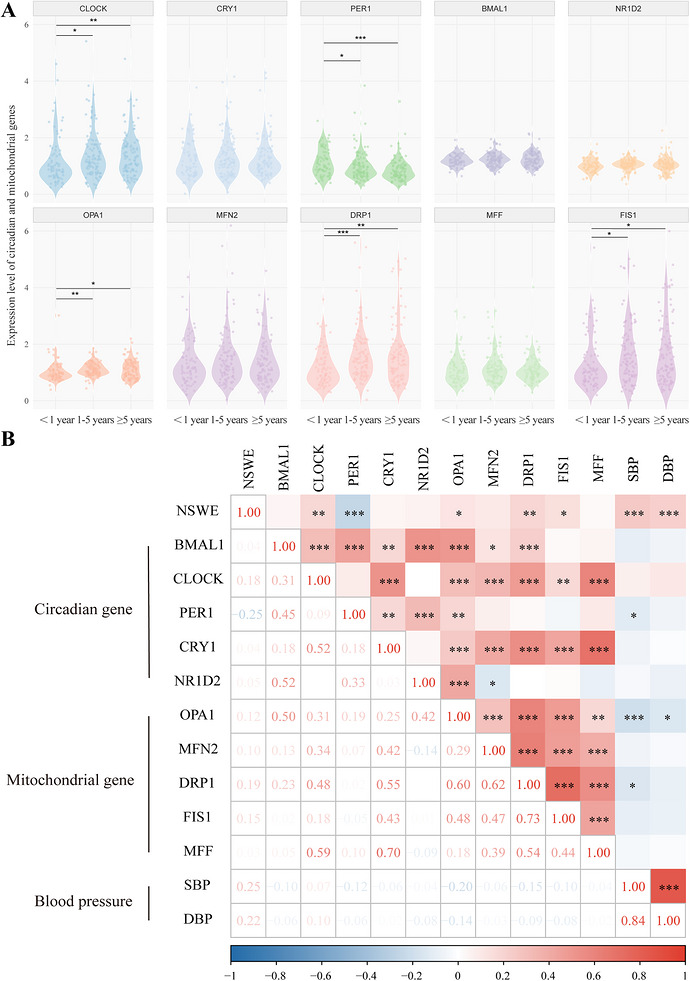
Relationship between night shift work exposure, circadian and mitochondrial gene expression, and blood pressure in humans. NSWE: duration of night shift work exposure; SBP: systolic blood pressure; DBP: diastolic blood pressure. (A) Circadian and mitochondrial gene expression at ZT0 by night shift work exposure duration. (B) Spearman correlation matrix among circadian genes, mitochondrial dynamics genes, night shift work exposure, and blood pressure. Red and blue indicate positive and negative correlations, respectively. ^*^ *p* < 0.05; ^**^ *p* < 0.01; ^***^ *p* < 0.001.

We next evaluated differential mRNA expression patterns of circadian and mitochondrial fission/fusion genes at different durations of night‐shift work exposure. Compared to workers with <1 year of night shift duration, workers with ≥5 years exhibited significantly higher expression levels of CLOCK (*p* = 0.006), OPA1 (*p* = 0.048), DRP1 (*p* = 0.004), and FIS1 (*p* = 0.032), but significantly lower levels of PER1 (*p* < 0.001, Figure 7A). The comprehensive relationships among night shift work duration, mRNA expression of circadian and mitochondrial markers, and blood pressure are presented in Figure 7B. Night shift work duration exhibited a significant positive correlation with *CLOCK* expression (Spearman's *ρ* = 0.18, *p* = 0.002), but a strong inverse association with *PER1* levels (Spearman's *ρ* = −0.25, *p* < 0.001). We also observed that night shift work duration was associated with the expression of mitochondrial regulatory genes (*OPA1*, *DRP1*, and *FIS1*) and directly correlated with blood pressure. Additionally, most circadian and mitochondrial genes exhibited inverse associations with blood pressure parameters. Specifically, *PER1* demonstrated a negative correlation with SBP (Spearman's ρ = −0.12, *p* = 0.037). Similarly, mitochondrial dynamics genes *OPA1* and *DRP1* showed significant inverse relationships with SBP (Spearman's *ρ* = −0.20 and −0.15, respectively; *p* < 0.001 and 0.013, respectively). And *OPA1* expression was also negatively associated with DBP (Spearman's *ρ* = −0.14, *p* = 0.018) (Figure 7B). Notably, we identified significant correlations between circadian gene expression patterns and mitochondrial fission/fusion regulators (Figure 7B), implying a potential regulatory role of circadian genes in mitochondrial dynamics.

## 3 Discussion

This study provides novel evidence implicating mitochondrial dysfunction as a potential mechanistic contributor to night shift work‐associated blood pressure elevation. Our rodent models demonstrated that simulated night‐shift work induces time‐dependent alterations in blood pressure. Population‐based findings further demonstrated an association between the duration of night shift work and elevated blood pressure. Most importantly, our study established the first animal experimental evidence demonstrating that simulated night‐shift work induces circadian disruption, mitochondrial dysfunction, and oxidative stress, with identifiable interrelationships among these pathological processes. These findings were further supported by a population‐based study that confirmed that night shift work exposure was associated with dysregulation of core circadian and mitochondrial biomarkers. Taken together, these findings indicate that night‐shift‐work‐induced circadian rhythm disruption exacerbates blood pressure elevation, with mitochondrial dysfunction emerging as the potential contributing mechanism.

While the existing evidence on the association between night shift work and blood pressure remains limited [17], this study incorporates both animal experimental and human population study designs to systematically examine this relationship. Our animal model revealed that after 10 weeks of simulated night shifts, systolic and diastolic blood pressure were significantly elevated in male mice at both ZT0 and ZT12, while in female mice, only systolic blood pressure was elevated at ZT0, with both parameters elevated at ZT12. Our population study found that workers with ≥5 years of night‐shift exposure exhibited a 10.012‐fold increased risk of elevated SBP and a 5.518‐fold increased risk of elevated DBP, consistent with previous findings [18, 19]. Furthermore, meta‐analysis showed that night shift work increases blood pressure, particularly among 24‐h shift workers [20], with the risk of blood pressure elevation rising in a frequency‐dependent manner [21].

To evaluate the potential role of circadian rhythm disruption from night shift work on blood pressure, we further examined clock gene expression in murine cardiac tissues and human peripheral blood. Night‐shift work simulation significantly perturbed circadian rhythms in mice. The arrhythmic patterns observed in CLOCK and PER1 may indicate lower‐amplitude oscillations in cardiac tissue relative to those in the liver or suprachiasmatic nucleus [22]. Our human results revealed identifiable correlations among night shift work exposure, clock gene expression profiles, and altered blood pressure. These findings align with an Italian study that reported similar correlations between disrupted circadian gene expression patterns and blood pressure dysregulation among night‐shift workers [5]. Further supporting evidence comes from an independent study that also documented circadian gene dysregulation in hypertensive patients [23]. However, the precise mechanism underlying circadian rhythm disruption remains incompletely understood. Given that the clock gene expression pattern is intrinsically regulated by circadian rhythms [24], our findings suggest that circadian misalignment induced by night shift work may play a mechanistic role in blood pressure elevation.

A critical knowledge gap persists regarding mitochondrial dysfunction as a potential mechanistic link between night shift work and elevated blood pressure. To address this gap, our animal experiments demonstrated that circadian disruption directly perturbs mitochondrial function, consistent with a previous study [25]. Complementing this, our population‐based study also showed that night shift work altered the expression levels of mitochondrial‐related markers [26]. Human data display more significant correlations between clock genes and mitochondrial markers than mouse data. This discrepancy likely reflects two factors. First, human participants were exposed to night‐shift work for longer periods than the 10‐week mouse model. Second, the larger human sample size increases statistical power to detect associations. Moreover, we observed a progressive decline in mitochondrial fusion activity from four to ten weeks of exposure. Critically, transmission electron microscopy and mitochondrial membrane potential analysis revealed the significant associations between clock gene expression and mitochondrial damage in murine cardiomyocytes. However, the detailed mechanisms underlying the association between circadian rhythm disruption and mitochondrial dysfunction remain to be investigated. Preliminary experimental evidence suggests the regulatory roles of circadian genes in mitochondrial dynamics [27]. Supporting this finding, another independent study demonstrates that knocking out the circadian gene can downregulate mitochondrial fission and mitophagy‐related genes, resulting in mitochondrial fragmentation and impaired mitophagy [28]. A potential mechanistic explanation is that core circadian clock genes are essential for maintaining normal mitochondrial function [14]. When these genes are mutated or dysregulated, mitochondrial dynamics may lose circadian rhythmicity, resulting in excessive ROS production and diminished mitochondrial membrane potential [27, 28, 29], and mitochondrial OXPHOS, with alterations in mitochondrial OXPHOS, biogenesis, and mitophagy levels [14, 30, 31].

Furthermore, our findings demonstrated an association between mitochondrial dysfunction and elevated blood pressure, aligning with previous research [32, 33]. Mitochondrial dysfunction, mediated by excessive ROS production, may represent a key mechanism contributing to elevated blood pressure [34, 35, 36]. Mechanistically, mitochondrial ROS induces angiotensin II (Ang II) production, thereby activating NADPH oxidase and ultimately promoting blood pressure elevation [37]. These findings suggest that mitochondrial dysfunction may be a key mechanistic link between night shift work and elevated blood pressure. Collectively, our integrated animal and human studies reveal a novel mechanism linking circadian clock profiles, mitochondrial dynamics, and blood pressure regulation. Specifically, circadian rhythm disruption caused by simulated night shift work affects mitochondrial fission and fusion, disrupting mitochondrial dynamics and, consequently, impairing normal mitochondrial function.

Our study identified significant sex differences in the observed relationships among circadian rhythm disruption, mitochondrial dysfunction, oxidative stress, and blood pressure elevation. Specifically, PER1 is significantly positively correlated with blood pressure in males, while negatively correlated with OPA1 and DRP1, implying that PER1 may contribute to blood pressure elevation by inhibiting mitochondrial dynamics. In contrast, in females, BMAL1 and CLOCK exhibit stronger correlations with blood pressure and mitochondrial markers, which may be attributed to estrogen's protective effects on mitochondrial function [38]. Beyond the direct protective role of estrogen, its interaction with the circadian rhythm further underlies the sex differences. Although estrogen possesses inherent antioxidant stress capacity [39], circadian rhythm disruption can interfere with its rhythmic secretion and increase androgen secretion [40]. This dysregulation of sex hormone secretion may subsequently reduce SOD synthesis, thereby impairing antioxidant capacity. Furthermore, an imbalance in sex hormone secretion can elevate oxidative stress levels and promote oxidative DNA damage [41, 42], exacerbating disruption of redox homeostasis. However, in‐depth cross‐sex analyses were restricted to Western blotting, as the 15‐well membrane's limited lane capacity precluded the simultaneous loading of male and female mouse samples on a single blot. Accordingly, male and female specimens were processed on separate membranes. Therefore, analyses were performed within each sex. This sex‐separated experimental design represents a common limitation in previous studies, highlighting the necessity of more rigorous designs for future research. Male and female samples will be processed side‐by‐side in the same experimental run to enable statistical analyses of interactions across sex, treatment and time points.

Notably, sex differences in oxidative stress susceptibility also contribute to this disparity. Males generally exhibit higher baseline oxidative stress levels than females [43], which may account for the decline in antioxidant stress capacity in male mice. Collectively, these findings indicate a comprehensive disruption of redox balance in the context of circadian rhythm disruption and mitochondrial dysfunction, and these sex differences are shaped by multiple interconnected factors. While estrogen is a key mediator of these effects, further research is warranted to clarify its specific role and the underlying molecular mechanisms.

Our study offers several key advances in understanding shift work‐related blood pressure elevation. First, we used a mouse model subjected to night‐shift work simulation, revealing that mice exposed to the simulated night‐shift condition had significantly elevated blood pressure. Second, integrating animal models and human studies, we confirmed that circadian disruption caused by night shift work adversely impacts blood pressure regulation. Most significantly, our results revealed the innovative role of mitochondrial dysfunction in this process, providing crucial mechanistic insights into pathophysiology. To the best of our knowledge, no previous study has demonstrated such an association in both occupational populations and animal models.

This study has several limitations. First, the relatively small sample size of the night‐shift worker cohort, coupled with the absence of data on smoking pack‐years, limits the generalizability of our findings. Future translational studies will address these shortcomings by integrating long‐term, dynamic monitoring in larger human populations, including the collection of detailed smoking histories. Second, the lack of circulating biomarkers from human samples constrains mechanistic translation from mouse to human; however, the primary aim of this study was to examine whether circadian disruption induced by night‐shift work could lead to elevated blood pressure through mitochondrial dysfunction. We have assessed mitochondrial gene expression in peripheral blood and found associations with blood pressure. Based on these findings, we will investigate the predictive value of mitochondrial markers for hypertension and incorporate measurements of cortisol, melatonin, and other circulating biomarkers. Third, although peripheral blood mononuclear cells (PBMCs)‐specific sampling is widely recognized as the preferred approach for circadian rhythm analysis, we instead analyzed clock gene expression in whole blood RNA. Such whole‐blood measurements inherently exhibit greater variability, primarily due to granulocyte contamination; this increased variability may reduce statistical power, yield smaller effect sizes, and limit the reproducibility of our clock gene expression results. Despite these technical constraints, our findings still provide preliminary population‐level insights into peripheral clock gene expression profiles in the study population. Fourth, clock‐related proteins were not co‐detected on the same blot, nor were mitochondrial targets analyzed on separate membranes with independent loading controls. We will co‐incubate clock‐associated targets with their respective loading controls on the same membrane and run separate blots for mitochondrial targets with independent loading controls. Finally, while the observed alterations in circadian and mitochondrial gene expression support the hypothesis that mitochondrial dysfunction may contribute to night‐shift‐associated hypertension, the findings are largely observational and lack sufficient depth to establish causality. Interventional studies, such as rescuing mitochondrial dynamics proteins (e.g., OPA1 over‐expression), are essential to determine whether circadian disruption‐induced hypertension is mediated through mitochondrial mechanisms.

## 4 Conclusion

Our findings demonstrate that night shift work is associated with elevated blood pressure in both human populations and mouse models, likely mediated by circadian rhythm disruption. Most significantly, this study reveals that mitochondrial dysfunction and elevated oxidative stress serve as key contributing mechanisms linking night shift work‐induced circadian rhythm disruption to blood pressure, providing valuable guidance for future research in cardiovascular diseases associated with circadian disruption.

## 5 Experimental Section/Methods

### 5.1 The Mouse Model of Night Shift Work Simulation

Adult C57BL/6J mice aged eight weeks were purchased from the Experimental Animal Center of Hangzhou Medical College. All mice were fed a standard chow diet (GB14924.3‐2010, Zhejiang Provincial Experimental Animal Center) with guaranteed nutritional values of ≥20.0% protein and ≥4.0% fat. The mice were randomly assigned to the simulated night‐shift work group (*n* = 50) and control group (*n* = 50). The number of male and female mice in both groups was equal. Then they were sacrificed at 4 weeks (*n* = 60) and 10 weeks (*n* = 40) separately. At four weeks, mice were sacrificed at five Zeitgeber Time (ZT) points: ZT0 (08:00 AM), ZT6 (02:00 PM), ZT12 (08:00 PM), ZT18 (02:00 AM, the next day), and ZT24 (08:00 AM, the next day), with six mice in each group at each ZT point. At 10 weeks, sampling was focused on ZT0 and ZT12, with 10 mice per group at each time point. Animals were housed with basic food and water at (26 ± 2°C) and (50 ± 5%) humidity and reared under a 12‐h light‐dark cycle environment.

All mice were allowed to adapt to the laboratory environment for one week. The control group was fed normally without interference in lighting conditions (light from 8:00 to 20:00, darkness from 20:00 to 8:00+1; 12 h light: 12 h dark per day). The mice in the night‐shift work simulation group were fed under interference in lighting conditions (light from 20:00 to 8:00+1, darkness from 8:00 to 20:00; 12 h light: 12 h dark per day). The light and dark conditions were reversed once a week. Mice were anesthetized using 3%–5% isoflurane and euthanized by cervical dislocation. After 4 weeks of night‐shift work simulation, heart tissues were collected at ZT0, ZT6, ZT12, ZT18, and ZT24 to investigate the effects of night‐shift work on circadian gene expression. All animal procedures were conducted in accordance with and approved by the Hangzhou Medical College Laboratory Animal Ethics Committee (2023‐060).

### 5.2 Measurement of Blood Pressure in Mice

Mice were subjected to a night‐shift work simulation for 4 or 10 weeks, and blood pressure was measured at ZT0 and ZT12 upon completion of the 4‐ and 10‐ week simulation. A noninvasive Blood Pressure System for Mice (BP‐2010A, Softron Biotechnology, Beijing, China) was used to measure blood pressure. The tail‐cuff method, combined with photoelectric plethysmography (PPG), was used. Mice were wrapped in a cloth or tube, and lubricant was applied to their tails. A tail‐cuff with an infrared sensor was placed on the tail. The cuff was inflated and then deflated to detect pulse waves and record pressure changes. The device's algorithms were used to calculate systolic blood pressure (SBP) and diastolic blood pressure (DBP). All mice underwent extensive habituation to the restraint and measurement procedure prior to data collection. Prior to each measurement session, mice were placed on a pre‐warmed platform for 10–15 min to allow physiological stabilization and vasodilation. Measurements were then taken in a quiet, temperature‐controlled room by the same experienced investigator. For each mouse at each time point, a minimum of 5 consecutive readings was recorded. The investigator was blinded to the group assignments.

### 5.3 Measurement of the Expression Levels of Genes Related to Circadian Rhythm and Mitochondrial Function in Mice

Heart samples were collected at two key circadian time points, ZT0 and ZT12, to assess diurnal variations. Mitochondrial morphology was examined using scanning electron microscopy (SEM). The apical portion of the mouse heart was excised and fixed in 2.5% glutaraldehyde. After using 0.1 m phosphate buffer, the heart samples were dehydrated at room temperature, osmotically embedded, then sectioned, stained, and finally examined using transmission electron microscopy. Quantitative assessment was performed by manual counting of mitochondria in captured images, with damaged mitochondria identified based on criteria including loss of cristae and rupture of mitochondrial membranes. To detect mitochondrial membrane potential in this study, a JC‐1 assay kit (Beyotime, Shanghai, China) was used. Following the manufacturer's protocol, mitochondria were isolated from fresh tissue and incubated with a 5‐fold‐diluted JC‐1 working solution (10–100 µg mitochondrial protein per 1 mL assay volume). The fluorescence intensity was monitored using a fluorescence microplate reader at excitation/emission wavelengths of 488/530 nm and 530/590 nm. Additionally, the protein expression levels of circadian rhythm and mitochondrial function‐related markers were assessed using a Western blot.

Circadian clock genes included circadian locomotor output cycles kaput (CLOCK), cryptochrome 1 (CRY1), period 1 (PER1), brain and muscle ARNT‐like 1 (BMAL1), and nuclear receptor subfamily 1 group D member 2 (NR1D2). Total RNA of heart tissues was isolated using TRIzol Reagent (Invitrogen). cDNA was synthesized using the PrimeScript RT Reagent Kit (Takara, Japan). Real‐time quantitative PCR was performed using a TB Green Premix Ex Taq II (Takara, Japan) and an ABI QuantStudio 7 qPCR system (Applied Biosystems, USA, California). The gene Glyceraldehyde‐3‐phosphate dehydrogenase (GAPDH) was used as a housekeeping gene and analyzed using the 2^(‐ΔΔCt) method. The primer sequences are listed in Table S1.

Additionally, the effect of night shift work on mitochondrial functional status was assessed by examining the expression levels of Optic Atrophy 1 (OPA1), Mitofusin 2 (MFN2), Dynamin‐Related Protein 1 (DRP1), Mitochondrial Fission 1 Protein (FIS1), NADH:Ubiquinone Oxidoreductase Subunit B8 (NDUFB8), Mitochondrially Encoded Cytochrome C Oxidase II (MTCO2), Microtubule‐Associated Protein 1 Light Chain 3 (LC3), and Sequestosome 1 (P62), PPAR γ Coactivator 1 (PGC‐1), Mitochondrial Transcription Factor A (TFAM). Isolate proteins from heart tissue using RIPA (Beyotime, Shanghai, China). The Pierce BCA Protein Assay Kit (Beyotime, Shanghai, China) was used to determine the protein content according to the manufacturer's protocol. Equal amounts of proteins were electrophoresed on 4–20% sodium dodecyl sulfate‐polyacrylamide gels (Yeasen, Shanghai, China) and then transferred to polyvinylidene difluoride membranes (PVDF; Merck Millipore, Germany). The membranes were closed with 5% skimmed milk and incubated with one of the following primary antibodies: anti‐PER1 (Proteintech, Wuhan, China), anti‐BMAL1 (Huabio, Hangzhou, China), anti‐CLOCK (Huabio, Hangzhou, China), anti‐DRP1 (Abcam, UK), anti‐OPA1 (Abcam, UK), anti‐MFN2 (Abcam, UK), anti‐FIS1 (Proteintech, Wuhan, China), anti‐NDUFB8 (Huabio, Hangzhou, China), anti‐MTCO2 (Abclonal, Wuhan, China), anti‐PGC‐1 (Huabio, Hangzhou, China), anti‐TFAM (Abclonal, Wuhan, China), anti‐LC3 (Cell Signaling Technology, USA), anti‐P62 (Huabio, Hangzhou, China), and anti‐GAPDH (Huabio, Hangzhou, China) at 4°C overnight. Information on all antibodies is shown in Table S2. After washing with TBST, the PVDF membrane was incubated with the secondary antibody HRP‐Conjugated Goat anti‐Rabbit IgG polyclonal Antibody (Huabio, Hangzhou, China). Protein bands on the PVDF membrane were visualized using a SuperKineTM Ultra Sensitive ECL Luminous Liquid (Abbkine Scientific, Wuhan, China) and quantified using Image J software.

### 5.4 Measurement of Oxidative Stress Levels in Mice

The supernatant from each heart sample collected at ZT0 and ZT12 was used to measure malondialdehyde (MDA), superoxide dismutase (SOD), Glutathione (GSH), and Glutathione Disulfide (GSSG) using commercial kits (Beyotime, Shanghai, China), according to the manufacturer's instructions. The GSH/GSSG ratio was calculated. And 8‐hydroxydeoxyguanosine (8‐OHdG) using commercial kits (Nanjing Jiancheng Bioengineering Institute, Nanjing, China),

### 5.5 Study Design of Population Study

According to data from a previous study among night shift workers [44], we calculated the expected sample size using a two‐sided significance level of 0.05, statistical power of 0.8, and a rate of loss to follow‐up (10%). Post‐hoc power analysis based on our observed BP means and standard deviations indicates that the present sample size provides >99% power to detect the observed effect sizes at α = 0.05. Accordingly, 347 participants were invited. The inclusion criteria for participants were: (i) age between 18 and 60 years; (ii) having signed the informed consent form. The exclusion criteria were: (i) individuals who are unable to cooperate with the investigation or refuse to participate in the investigation; (ii) workers whose family had a history of hypertension; (iii) no occupational information was provided; (iv) lack of occupational history of night shift work. Finally, 68 were excluded due to the lack of occupational history of night shift work, and 279 were included in the study. This study was approved by the Ethics Committee of Hangzhou Medical College (LL2023‐03).

### 5.6 Questionnaire Survey and Physical Examination

Participants signed informed consent forms when donating peripheral venous blood and completed a questionnaire to collect information on anthropometric data, educational level, smoking habits, alcohol drinking habits, and night‐shift work exposure. The education level was classified as less than high school or above [45]. Smoking habit was classified as no and yes (including former smokers and current smokers) [46]. Drinking habit was classified as no and yes (including occasional drinking and regular drinking) [47]. Anthropometric information, including weight and height, was obtained using rangefinders and electronic weight scales [48]. Body Mass Index (BMI) was calculated based on height and weight and categorized into normal and overweight using 24 kg/m^2^ as the cutoff point [49].

### 5.7 Night Shift Work Assessment in the Population

Shift work was defined as “a work schedule that extends beyond the normal daytime working hours from 9 a.m. to 5 p.m”, and night work was specifically defined as “a work schedule that involves working during normal sleeping hours, such as within the working period from 12 a.m. to 6 a.m” [3, 50]. Shift work was categorized as “no shift work”, “day work”, “night shift work”, and “night work” (Table S3). Participants employed at the time of recruitment were considered for night shift work. Participants provided information on the year they started their previous night shift work, and we calculated the exposure to night shift work (i.e., the number of years working night shifts). The workers were grouped by their exposure duration to night shift work: <1 year, 1–4 years, and ≥5 years.

### 5.8 Measurement of Blood Pressure in the Population

All study subjects had their blood pressure checked by a specialized physician at 08:00 a.m. (ZT0) after 15 min of seated rest, 12 h after being removed from the work environment. The diagnostic criteria for hypertension are systolic blood pressure (SBP) ≥ 140 mmHg and/or diastolic blood pressure (DBP) ≥ 90 mmHg on two different days [51].

### 5.9 Measurement of Gene Expression Levels Related to Circadian Rhythm and Mitochondrial Function in the Population

Blood samples were collected between 07:30 and 08:30 h (ZT0) after overnight fasting and 15 min of seated rest. Total RNA was isolated from blood using the TRIzol LS Reagent Blood/Liquid Sample RNA Extraction Kit (BioTeke, Beijing, China) [52]. cDNA was synthesized using the PrimeScript RT Kit (Takara, Japan). Real‐time quantitative polymerase chain reaction was performed using TB Green Premix Ex Taq II (Takara, Japan) and the ABI QuantStudio 7 qPCR System (Applied Biosystems, California, USA). The gene GAPDH was used as the housekeeping gene and analyzed using the 2^−ΔΔCt^ method. The primer sequences are listed in Table S1.

### 5.10 Statistical Analysis

Statistical analysis was conducted using RStudio 4.2.0 software. Continuous variables were described using the median (*M*) and the 25th and 75th percentiles (*P*_25_, *P*_75_). For comparisons between two groups, independent‐samples *t*‐tests were used when the data followed a normal distribution; otherwise, the Mann‐Whitney *U* test was applied. For comparisons among multiple groups in human subjects, analysis of variance (ANOVA) was used if the data were normally distributed. Otherwise, the Kruskal‐Wallis *H* test and Dunn's post hoc test were employed. Data from experimental mice were analyzed using two‐way ANOVA models with treatments and time points as factors. Initial full factorial models were constructed to include all main effects and interaction terms. For outcomes with significant interaction effects, stratified comparisons of treatment groups were performed at each time point using EMM comparisons. For outcomes without significant interactions, interaction terms were removed, and main‐effects models were used to compare the two treatment groups. Pearson's correlation coefficients were used for normally distributed variables, while Spearman's rank‐order correlation coefficients were used for non‐normally distributed data. The comparison of circadian rhythms between the two groups was performed by the non‐linear regression method using the CircaCompare R package [53]. Rhythmicity was defined as a COSINOR fit with *p* < 0.05. Genes that failed this test are indicated by a dashed line instead of a fitted cosine curve. Multiple linear regression was used to assess the association of night‐shift work exposure with blood pressure, adjusting for confounding factors. Multiple linear regression analyses were conducted to analyze the association between night shift work and blood pressure, using gender, age, education level, BMI, smoking habit, and drinking habit as controlling variables. *p* ≤ 0.05 was considered statistically significant.

## Author Contributions

Z. J. and Y. D. participated in experimental design, data collection and analysis, manuscript drafting, and execution of experiments. Y. L. provided critical experimental materials and technical guidance. S. L. and J.P. L. performed experimental operations and validation. H. X. and L. F. conducted data processing and statistical analysis. Y. L., J. Z., and C. Z. conducted the literature review and prepared the figures. T. L., J. W., X. W., L. L., and J. Z. participated in sample collection and evaluation. X. Z. provided financial support. J. L. was responsible for overall research planning, manuscript revision, and final approval.

## Conflicts of Interest

The authors declare no conflict of interest.

## Supporting information




**Supporting File**: advs76212‐sup‐0001‐SuppMat.docx.

## Data Availability

The data that support the findings of this study are available from the corresponding author upon reasonable request.
