# Supplementary material for: Mitochondrial Dysfunction Unravels the Potential Molecular Link Between Night Shift Work‐Related Circadian Disruption and Elevated Blood Pressure in Human and Mouse Models
Source: Adv Sci (Weinh). 2026 Jun 19:e20318. Online ahead of print. doi: 10.1002/advs.202520318 (PMC13336779; doi:10.1002/advs.202520318)
Supplement: Supplementary file 1 — Supporting File: advs76212‐sup‐0001‐SuppMat.docx. [file ADVS-9999-e20318-s001.docx]

**Supporting Information**

**Mitochondrial dysfunction unravels the potential molecular link between night shift work-related circadian disruption and elevated blood pressure in human and mouse models**

Zhaoqiang Jiang, Yifan Dou, Yingnan Lei, Shuang Liu, Jialin Zhou, Jiaping Li, Huadong Xu, Lingfang Feng, Yongxin Li, Chuyan Zhang, Tao Li, Jianfei Wang, Xing Zhang, Xinran Wang, Luyao Liu, Jingjing Zhang, Jianlin Lou*.

This PDF file includes:

Supplementary Figure 1–5.

Supplementary Tables 1–3.


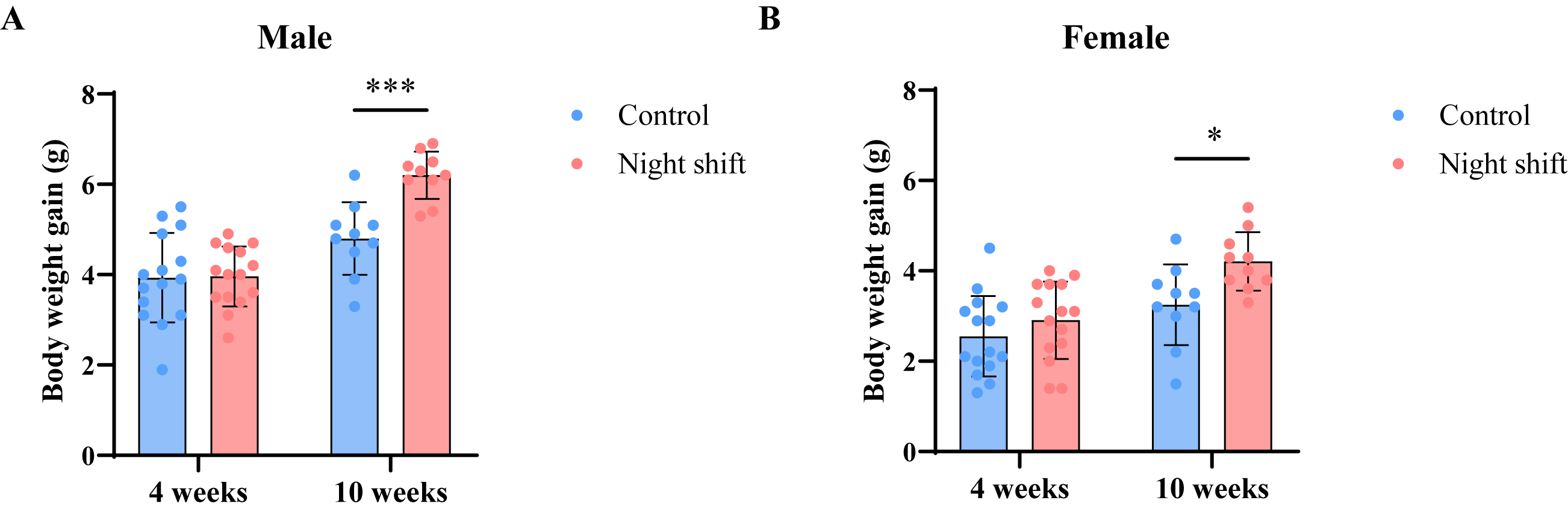


**Supplementary Figure 1.** The impact of night-shift work simulation on body weight gain in mice at 4 weeks (n = 15) and 10 weeks (n = 10). Night shift: night shift work simulation. A) male. B) female. **P* < 0.05; ****P* < 0.001.

**
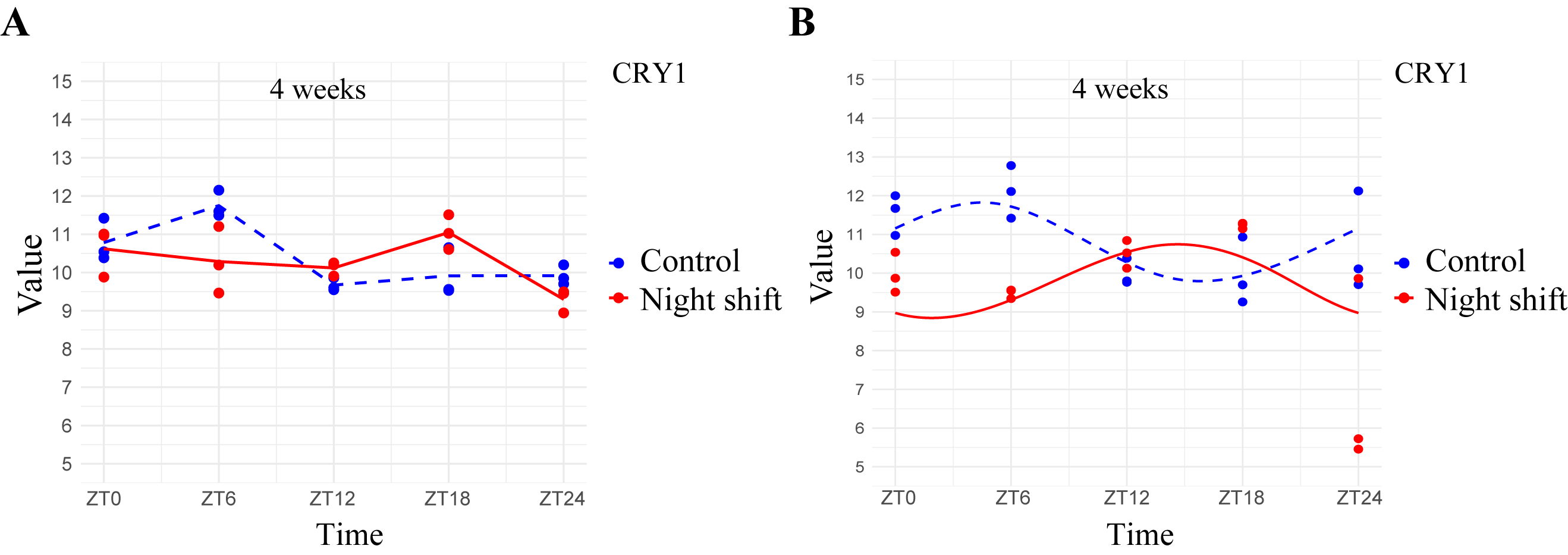
**

**Supplementary Figure 2.** The mRNA expression of CRY1 in the myocardium of mice for 4 weeks simulated night-shift at 5 time points (n = 3 per time point). A) male; B) female. Night shift: night shift work simulation. Rhythmicity was assessed using the CircaCompare test with α = 0.05. Neither male nor female data exhibited significant rhythmicity; amplitude and acrophase are therefore not presented.

**Supplementary Figure 3.** Circadian disruption and mitochondrial dysfunction induced by night shift work simulation. A–B) Expression of circadian marker proteins in male (A) and female (B) mice at ZT0 and ZT12 after 10 weeks of simulated night shift exposure vs. control group (n = 2). C–D) Expression of mitochondrial marker proteins in male (C) and female (D) mice at ZT0 and ZT12 after 10 weeks of simulated night shift exposure vs. control group (n = 2). E–F) Expression of mitochondrial marker proteins in male (E) and female (F) mice at ZT0 and ZT12 after 10 weeks of simulated night shift exposure vs. control group (n = 2). G–H) Expression of mitochondrial marker proteins in male (G) and female (H) mice at ZT0 and ZT12 after 10 weeks of simulated night shift exposure vs. control group (n = 2). I–J) Expression of mitochondrial marker proteins in male (I) and female (J) mice at ZT0 and ZT12 after 10 weeks of simulated night shift exposure vs. control group (n = 2).

**Supplementary Figure 4.** Original Western blot images. The red numbers (-1 and -2) in the figure indicate the incubation order.

**Supplementary Figure 5.** Mitochondrial dysfunction induced by night shift work simulation. A–B) Expression of mitochondrial marker proteins in male (A) and female (B) mice at ZT0 and ZT12 after 4 weeks (n = 3) of simulated night shift exposure vs. control group.

C–D) Expression of mitochondrial marker proteins in male (C) and female (D) mice at ZT0 and ZT12 after 10 weeks (n = 3) of simulated night shift exposure vs. control group.

E–F) Expression of mitochondrial marker proteins in male (E) and female (F) mice at ZT0 and ZT12 after 4 weeks (n = 3) of simulated night shift exposure vs. control group.

G–H) Expression of mitochondrial marker proteins in male (G) and female (H) mice at ZT0 and ZT12 after 10 weeks (n = 3) of simulated night shift exposure vs. control group.

I–J) Expression of mitochondrial marker proteins in male (I) and female (J) mice at ZT0 and ZT12 after 4 weeks (n = 3) of simulated night shift exposure vs. control group.

K–L) Expression of mitochondrial marker proteins in male (K) and female (L) mice at ZT0 and ZT12 after 10 weeks (n = 3) of simulated night shift exposure vs. control group.

Data presented as mean ± SD. Statistical analysis was performed using two-way ANOVA followed by EMM comparisons. * *P* < 0.05; ** *P* < 0.01; ****P* < 0.001.

**Supplementary Table 1.** The sequences of the PCR primer of genes related to circadian rhythm and mitochondrial function.

| Gene name | Primer | Sequence |
| --- | --- | --- |
| GAPDH-F (human) | Forward primer 5’-3’ | GAGAAGGCTGGGGCTCATTT |
| GAPDH-R (human) | Forward primer 5’-3’ | AGTGATGGCATGGACTGTGG |
| BMAL1-F (human) | Forward primer 5’-3’ | AAGGGAAGCTCACAGTCAGAT |
| BMAL1-R (human) | Forward primer 5’-3’ | GGACATTGCGTTGCATGTTGG |
| CLOCK-F (human) | Forward primer 5’-3’ | AAGTTAGGGCTGAAAGACGACG |
| CLOCK-R (human) | Forward primer 5’-3’ | GAACTCCGAGAAGAGGCAGAAG |
| PER1-F (human) | Forward primer 5’-3’ | CGATGCCAACAGCAATGGTT |
| PER1-R (human) | Forward primer 5’-3’ | GGGCTCTGAGAGTTTGTGCT |
| CRY1-F (human) | Forward primer 5’-3’ | CCTGGAATGCACCAGAAGGT |
| CRY1-R (human) | Forward primer 5’-3’ | CCATGGAGCTTCTTGCTTCAAC |
| NR1D2-F (human) | Forward primer 5’-3’ | TTTACACTCAATGGGAGCAGGG |
| NR1D2-R (human) | Forward primer 5’-3’ | TGCAGATACCAGGACAACAGC |
| MFN2-F (human) | Forward primer 5’-3’ | CTCTCGATGCAACTCTATCGTC |
| MFN2-R (human) | Forward primer 5’-3’ | TCCTGTACGTGTCTTCAAGGAA |
| OPA1-F (Human) | Forward primer 5’-3’ | TGTGAGGTCTGCCAGTCTTTA |
| OPA1-R (human) | Forward primer 5’-3’ | TGTCCTTAATTGGGGTCGTTG |
| DRP1-F (human) | Forward primer 5’-3’ | TTTGACACTTGTGGATTTGCCA |
| DRP1-R (human) | Forward primer 5’-3’ | AGTGACAGCGAGGATAATGGA |
| MFF-F (human) | Forward primer 5’-3 | AACCCCTGGCACTGAAAACA |
| MFF-R (human) | Forward primer 5’-3 | TGCCAACTGCTCGGATTTCT |
| FIS1-F (human) | Forward primer 5’-3’ | GATGACATCCGTAAAGGCATCG |
| FIS1-R (human) | Forward primer 5’-3’ | AGAAGACGTAATCCCGCTGTT |
| GAPDH-F (mice) | Forward primer 5’-3’ | GAGAAGGCTGGGGCTCATTT |
| GAPDH-R (mice) | Forward primer 5’-3’ | AGTGATGGCATGGACTGTGG |
| BMAL1-F (mice) | Forward primer 5’-3’ | AAGGGAAGCTCACAGTCAGAT |
| BMAL1-R (mice) | Forward primer 5’-3’ | GGACATTGCGTTGCATGTTGG |
| CLOCK-F (mice) | Forward primer 5’-3’ | AAGTTAGGGCTGAAAGACGACG |
| CLOCK-R (mice) | Forward primer 5’-3’ | GAACTCCGAGAAGAGGCAGAAG |
| PER1-F (mice) | Forward primer 5’-3’ | CGATGCCAACAGCAATGGTT |
| PER1-R (mice) | Forward primer 5’-3’ | GGGCTCTGAGAGTTTGTGCT |
| CRY1-F (mice) | Forward primer 5’-3’ | CCTGGAATGCACCAGAAGGT |
| CRY1-R (mice) | Forward primer 5’-3’ | CCATGGAGCTTCTTGCTTCAAC |
| NR1D2-F (mice) | Forward primer 5’-3’ | TTTACACTCAATGGGAGCAGGG |
| NR1D2-R (mice) | Forward primer 5’-3’ | TGCAGATACCAGGACAACAGC |

**Supplementary Table 2.** The antibodies targeting proteins related to circadian rhythm and mitochondrial function.

| Antibody | Reference number | Company | Species |
| --- | --- | --- | --- |
| Anti-PER1 | 13463-1-AP | Proteintech | Rabbit |
| Anti-BMAL1 | ET1705-5 | Huabio | Rabbit |
| Anti-CLOCK | 82829-1-RR | Proteintech | Rabbit |
| Anti-DRP1 | AB184247 | Abcam | Rabbit |
| Anti-OPA1 | AB157457 | Abcam | Rabbit |
| Anti-MFN2 | AB124773 | Abcam | Rabbit |
| Anti-FIS1 | 10956-1-AP | Proteintech | Rabbit |
| Anti-NDUFB8 | ET7108-25 | Huabio | Rabbit |
| Anti-MTCO2 | A11522 | Abclonal | Rabbit |
| Anti-LC3 Ⅰ/Ⅱ | 12741T | Cell signaling technology | Rabbit |
| Anti-P62 | PS00-61 | Huabio | Rabbit |
| Anti-PGC-1 | ET1702-96 | Huabio | Rabbit |
| Anti-TFAM | A13552 | Abclonal | Rabbit |
| Anti-GAPDH | R1210-1 | Huabio | Rabbit |
| Anti-Rabbit IgG polyclonal | HA1001 | Huabio | Anti-Rabbit |

**Supplementary Table 3**. Job categories of all participants upon enrollment.

| Question 1: Does your work involve shift work? | Question 2: Does your job involve night shifts? | Shift work type |
| --- | --- | --- |
| Never/rarely | - | No shift work |
| Sometimes | Never/rarely | Day work |
|  | Sometime | Night shift work |
|  | Usually | Night shift work |
|  | Always | Night shift work |
| Usually | Never/rarely | Day work |
|  | Sometime | Night shift work |
|  | Usually | Night shift work |
|  | Always | Night shift work |
| Always | Never/rarely | Day work |
|  | Sometime | Night shift work |
|  | Usually | Night shift work |
|  | Always | Night work |
